# Novel Dextran Coated Cerium Doped Hydroxyapatite Thin Films

**DOI:** 10.3390/polym14091826

**Published:** 2022-04-29

**Authors:** Carmen Steluta Ciobanu, Ionela Cristina Nica, Anca Dinischiotu, Simona Liliana Iconaru, Patrick Chapon, Bogdan Bita, Roxana Trusca, Andreea Groza, Daniela Predoi

**Affiliations:** 1 National Institute of Materials Physics, Atomistilor Street, No. 405A, P.O. Box MG 07, 077125 Magurele, Romania; ciobanucs@gmail.com (C.S.C.); simonaiconaru@gmail.com (S.L.I.); 2 Department of Biochemistry and Molecular Biology, Faculty of Biology, University of Bucharest, 91-95 Splaiul Independentei, 050095 Bucharest, Romania; cristina.nica@drd.unibuc.ro (I.C.N.); anca.dinischiotu@bio.unibuc.ro (A.D.); 3 Research Institute of the University of Bucharest–ICUB, University of Bucharest, 050657 Bucharest, Romania; 4 HORIBA Jobin Yvon S.A.S., 6-18, Rue du Canal, CEDEX, 91165 Longjumeau, France; patrick.chapon@horiba.com; 5 National Institute for Laser, Plasma and Radiation Physics, 409 Atomistilor Street, P.O. Box MG 36, 077125 Bucharest, Romania; bogdan.bita@inflpr.ro; 6 Department of Science and Engineering of Oxide, Faculty of Applied Chemistry and Materials Science, Materials and Nanomaterials, University “Politehnica” of Bucharest, 060042 Bucharest, Romania; truscaroxana@yahoo.com; 7 Centre for Micro and Nanomaterials, University “Politehnica” of Bucharest, 060042 Bucharest, Romania

**Keywords:** dextran coated cerium doped hydroxyapatite, composite coatings, surface morphology, chemical composition, biocompatibility, human gingival fibroblast cells

## Abstract

Dextran coated cerium doped hydroxyapatite (Ca_10-x_Cex(PO_4_)_6_(OH)_2_), with x = 0.05 (5CeHAp-D) and x = 0.1 (10CeHAp-D) were deposited on Si substrates by radio frequency magnetron sputtering technique for the first time. The morphology, composition, and structure of the resulting coatings were examined by scanning electron microscopy (SEM), energy-dispersive x-ray spectroscopy (EDX), atomic force microscopy (AFM), metallographic microscopy (MM), Fourier transform infrared spectroscopy (FTIR), and glow discharge optical emission spectroscopy (GDOES), respectively. The obtained information on the surface morphologies, composition and structure was discussed. The surface morphologies of the CeHAp-D composite thin films are smooth with no granular structures. The constituent elements of the CeHAp-D target were identified. The results of the FTIR measurements highlighted the presence of peaks related to the presence of ν_1_, ν_3_, and ν_4_ vibration modes of (PO_4_^3−^) groups from the hydroxyapatite (HAp) structure, together with those specific to the dextran structure. The biocompatibility assessment of 5CeHAp-D and 10CeHAp-D composite coatings was also discussed. The human cells maintained their specific elongated morphology after 24 h of incubation, which confirmed that the behavior of gingival fibroblasts and their proliferative capacity were not disturbed in the presence of 5CeHAp-D and 10CeHAp-D composite coatings. The 5CeHAp-D and 10CeHAp-D coatings’ surfaces were harmless to the human gingival fibroblasts, proving good biocompatibility.

## 1. Introduction

The production and characterization of hydroxyapatite materials in various chemical structures is a promising research topic, due to the medical and biological applications of these compounds. Natural hydroxyapatite (HAp) is the major mineral constituent extensively present in bones and teeth (enamel and dentine) and is largely used in reconstruction engineering. It can contain cations such as Mg^2+^ and Na^2+^, or anions such as CO_3_^2−^ and OH^−^ [1,2]. Nevertheless, synthetic hydroxyapatite Ca_10_(PO_4_)_6_(OH)_2_ is widely used for the repair and regeneration of bone hard tissue, considering its biocompatibility, non-toxicity and osteoconductive properties [1,2,3,4]. Hydroxyapatite can also encourage the growth and adherence of osteoblast and osteoclast cells, as well as their proliferation [3,4].

The crystalline lattice of HAp contains PO_4_^3−^ tetrahedron groups linked to Ca^2+^ and OH^−^ groups [1,2]. The distance between PO_4_^3−^ groups is large, and therefore, different cations or anions can be accommodated in the crystalline structure [1,5,6]. Anion substitutions of OH^−^ with F^−^ or Cl^−^ ions can occur in the apatite structure. Moreover, the cation substitution of Ca^2+^ can be performed with monovalent (Ag^+^, Na^+^, K^+^), bivalent (Mg^2+^, Sr^2+^, Zn^2+^, Ba^2+,^ Cu^2+^) or trivalent (Ce^3+^, Sm^3+^, Eu^3+^) ions [5,6,7,8].

The cation substitution of Ca^2+^ ions in the apatite lattice provides novel and improved properties to the doped hydroxyapatite materials, such as biocompatibility, bioactivity, non-toxicity, reduced bone fragility, and cell proliferation or growth [1,5,6,7,8]. In addition, as coatings, doped hydroxyapatite can promote interfacial bonding with implants, prostheses, or surrounding tissue. Na-doped HAp, or Sr-doped HAp can promote osteoblast and stem cell proliferation while the Zn-doped HAp and Mg-doped HAp can supplementarily increase the viability, adhesion, or spread of cells [1,2].

The substitution of Ca^2+^ ions with trivalent ions of rare-earth elements, such as Ce^3+^, Sm^3+^, and Eu^3+^, in the HAp crystalline structure is very suitable, as their radii are very similar [1,7], even if the cytotoxicity of the synthetized compounds is still an open issue [1,9]. For 5% atomic concentrations, the doping of HAp powder by the co-precipitation method at lower doses of 100 micrograms/mL showed no significant cytotoxicity against mouse L929 fibroblast cells [10]. In addition, cerium can substitute calcium, increase the metabolism of bones, antimicrobial activity, as well as bone regeneration [1,9,10,11]. However, several studies indicated that the cytotoxicity, biocompatibility, and antimicrobial activity of Ce doped HAp are dependent on the synthesis method and dopant concentration [1,9,10,11].

When applied as coatings to cover medical implants or orthopedic prostheses, doped hydroxyapatite layers can improve the medical devices’ properties by promoting strong interfacial bonds, improved biocompatibility, and antimicrobial activity. Several phyisco-chemical techniques are now used for the production of such layers: electrochemical deposition [12], micro-arc oxidation [13], sol-gel method [14], and biomimetic deposition [15]. In addition, plasma spray, magnetron sputtering discharges, pulsed laser [16,17] and electron deposition are methods used for the fabrication of doped hydroxyapatite layers.

Moreover, in order to improve the physicochemical and biological properties of such layers, biocompatible and nontoxic polysaccharides, such as dextran (H(C_6_H_10_O_5_)_x_OH), are often used [18]. Among various applications of dextran, we mention wastewater treatment [19], drug delivery, antithrombotic agent, tissue engineering, [20], blood supplement [18], etc. In the study conducted by Hussain, M.A. et al. [21] it has been shown that dextran can be used as a stabilizing agent for silver nanoparticles. In the paper elaborated by Predoi, D. and collaborators [22], it has been highlighted that the presence of dextran on zinc doped hydroxyapatite nanoparticles’ surface does not alter their biological properties.

Furthermore, in our previous paper [7], we showed that Ce-doped HAp powders (produced by a co-precipitation method) can be used as a sputtering target for generation in radio frequency magnetron sputtering discharges of Ce-doped HAp layers with antimicrobial properties against gram-positive *Staphylococcus aureus* ATCC 25923, gram-negative *Escherichia coli* ATCC 25922, and fungal strain *Candida albicans ATCC 9002*. However, we generated, by radio frequency magnetron sputtering technique, a high diversity of HAp based coatings on metallic or polymeric substrates [7,8,23,24].

In the present work, we report, for the first time, our results regarding the morphology, composition, structure, and biocompatibility of dextran coated cerium doped hydroxyapatite (Ca_10-x_Cex(PO_4_)_6_(OH)_2_) thin films, with x = 0.05 (5CeHAp-D) and x = 0.1 (10CeHAp-D), obtained by radio frequency magnetron sputtering technique. This study demonstrates, for the first time, that the human gingival fibroblast cells retain their morphology in the presence of CeHAp-D composite coatings. Furthermore, this research showed, for the first time, that the behavior of gingival fibroblasts and their proliferative capacity were not disturbed in the presence of CeHAp-D composite coatings.

## 2. Materials and Methods

### 2.1. Materials

The powders of dextran coated cerium doped hydroxyapatite Ca_10−x_Ce_x_(PO_4_)_6_(OH)_2_, with x = 0.05 (5CeHAp-D) and x = 0.1 (10CeHAp-D), were obtained using calcium nitrate tetrahydrate Ca(NO_3_)_2_∙4H_2_O (Sigma Aldrich, St. Louis, MO, USA, ≥99.0%), cerium nitrate hexahydrate Ce(NO_3_)_2_·6H_2_O (Alfa Aesar ThermoFisher GmbH Company, Kandel, Germany, 99.97% purity), (NH_4_)_2_HPO_4_ (Sigma Aldrich, St. Louis, MO, USA, ≥99.0%), ammonium hydroxide NH_4_OH [Sigma Aldrich, St. Louis, MO, USA, 25% NH_3_ in H_2_O (T)], dextran (H(C_6_H_10_O_5_)_x_OH, (MW ~ 40,000), Merck, Kenilworth, NJ, USA), ethanol absolute, and double distilled water.

### 2.2. Synthesis of Dextran Coated Cerium Doped Hydroxyapatite (CeHAp-D) Powders

The powders of dextran coated cerium doped hydroxyapatite Ca_10−x_Ce_x_(PO_4_)_6_(OH)_2_, with x = 0.05 (5CeHAp-D) and x = 0.1 (10CeHAp-D), were obtained in agreement with our previous studies [22] where the (Ca+Ce)/P molar ratio was 1.67. The powders were obtained through an adapted sol-gel method in ambient conditions. For this purpose, two solutions were obtained by dissolving the initial precursors in ethanol absolute. Therefore, a solution was obtained by dissolving the appropriate amount of (NH_4_)_2_HPO_4_ in ethanol absolute. Secondly, Ca(NO_3_)_2_·4H_2_O and Zn(NO_3_)_6_·6H_2_O were dissolved in distilled water under vigorous agitation by a magnetic stirrer. The obtained solutions were dropped in a dextran solution. The resulting solution was stirred at 100 °C in order to obtain a gel. Then, the gel was washed several times with ethanol and double-distilled water [22]. The procedure is described in detail elsewhere [22].

### 2.3. Preparation of Dextran Coated Cerium Doped Hydroxyapatite Layers by Magnetron Sputtering Technique

The dextran coated cerium doped hydroxyapatite composite layers were deposited on silicon substrates by radio frequency (RF) magnetron sputtering technique. Firstly, sputtering targets of 5CeHAp-D and 10CeHAp-D in cylindrical shapes were obtained after the cold pressing of powders for a few minutes in air at atmospheric pressure. The geometrical sizes of the sputtering targets are the following: 2 mm thick, and 50 mm in diameter. Then, in turn, each sputtering target was placed inside the magnetron source for deposition of 5CeHAp-D and 10CeHAp-D layers, respectively. The target erosion zone had a diameter of about 4 cm.

**Figure 1:**
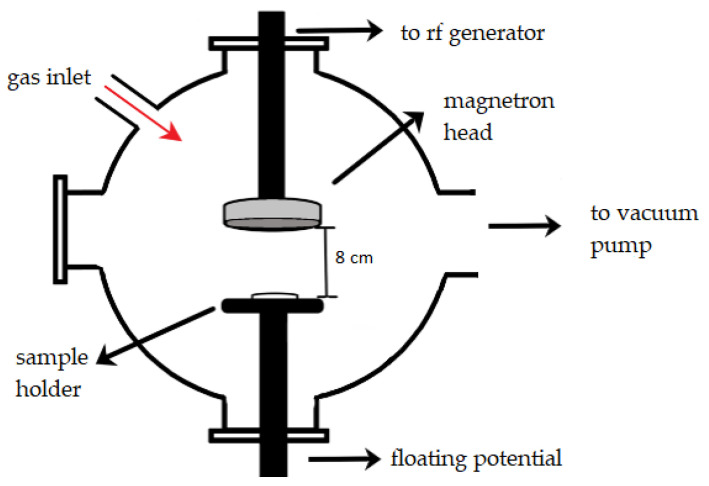
Experimental set-up.

The experimental setup is presented in Figure 1, and the deposition procedure was previously presented in detail in [23,24].

The magnetron source (acquired from K.J. Lesker Company, Hastings, UK) was coupled to a 13.56 MHz RF source through a matching box (Dressler, Cesar 136, Advanced Energy Industries GmbH & AEI Power GmbH, Metzingen, Germany). The substrate holder was at floating potential without being heated or cooled. Both types of layers were generated in the following experimental conditions: 50 W RF power, 180 min deposition time, 4 × 10^−3^ mbar Ar gas pressure (base pressure ~10^−5^ mbar), and 6 mln/min gas flow. For the 50.24 cm^2^ plasma exposure area of the sputtering target, the power density was about 3.9 W/cm^2^. 

By applying RF electrical power to the magnetron source, the target was is struck and sputtered mainly by the Ar^+^ ions produced in the plasma. Further, the atoms and ions sputtered from the target are deposited as layers on Si surfaces positioned on the sample holder centrally to the magnetron source head. The surfaces of the silicon substrates (5 mm × 5 mm × 1 mm) were optically polished. By maintaining the substrate holder at floating potential during the entire deposition process, a significant sputtering of the film, due to ion bombardment, was avoided. 

The thicknesses of the layers were calculated by measuring the deposition rates of 5CeHAp-D and 10CeHAp-D in the RF magnetron discharge. The quartz microbalance (acquired from INFICON Company, Overland Park, KS, USA) positioned on the substrate holder centrally to the magnetron source measured a deposition rate of about ~0.1 Å/s (with a deviation of ~0.01 Å/s). The calculated thickness of the layers was about ~110 nm. The doping of hydroxyapatite with different concentrations of Ce ions did not significantly influence the values of the deposition rates for 5CeHAp-D and 10CeHAp-D layers, respectively. The temperature of the substrate holder was measured during the plasma depositions using a thermocouple probe. Thus, 10 min after plasma ignition, the temperature at of the surfaces of Si substrates was ~100, and after 3 h reached 135 °C.

### 2.4. Physico-Chemical Characterisations

The microstructure of 5CeHAp-D and 10CeHAp-D composite layers was characterized by scanning electron microscopy (SEM) using an FEI Inspect S scanning electron microscope (Hillsboro, OR, USA), in both high- and low-vacuum modes. The EDAX Inc. SiLi detector attached to the microscope allowed precise analysis of the elemental composition of the films by energy dispersive X-ray spectroscopy (EDS). The dispersive energy X-ray (EDS) spectra of 5CeHAp-D and 10CeHAp-D composite layers as well as the mapping of their elemental distribution have been recorded at 10 kV applied on the field emission gun. The Studies on the element distributions of 5CeHAp-D and 10CeHAp-D composite thin films were performed using glow discharge optical emission spectroscopy (GDOES) (Horiba Company, Longjumeau, France) [25]. The identification of functional groups, characteristic of 5CeHAp-D and 10CeHAp-D composite thin film structures, was performed by Fourier transform infrared spectroscopy (FTIR) studies. The FTIR measurements were performed with the help of a Perkin Elmer SP-100 spectrometer (Waltham, MA, USA). For these studies, the equipment was used in ATR mode. Moreover, all the FTIR spectra were collected in the spectral region of 550–4000 cm^−1^,with a scan resolution of 4 cm^−1^. In addition, in agreement with the procedure described in detail in [26], the second derivative spectra of CeHAp-D composite layers were obtained.

Supplementary information regarding the morphology and roughness (root mean square roughness (R_RMS_) parameter) of the 5CeHAp-D and 10CeHAp-D composite thin films were obtained by atomic force microscopy (AFM) analysis. An NT-MDT NTEGRA probe nano laboratory instrument (NT-MDT, Moscow, Russia), operated in semi-contact mode (using a silicon NT-MDT NSG01 cantilever (NT-MDT, Moscow, Russia) coated with a 35 nm gold layer), was used in order to obtain information about the surface topography of the samples. The atomic force microscopy images were obtained with a on surface areas of 5 × 5 µm^2^. The collected data were analyzed with Gwyddion 2.59 software (Department of Nanometrology, Czech Metrology Institute, Brno, Czech Republic) [27]. 

The surface morphology of the obtained thin films was also studied with the help of the metallographic microscopy (MM) technique. Therefore, the MM studies were performed with the help of an inversed trinocular metallographic microscope OX.2153-PLM, (Euromex, Arnhem, The Netherlands). The MM images were obtained with a CMEX digital camera using the 10X objective of the microscope and ImageFocus Alpha software. The 3D representations of the MM images were obtained with the aid of ImageJ software [28]. 

### 2.5. Biological Evaluations

#### 2.5.1. Culture of HGF-1 Fibroblasts on CeHAp-D Composite Coatings

**Figure 2:**
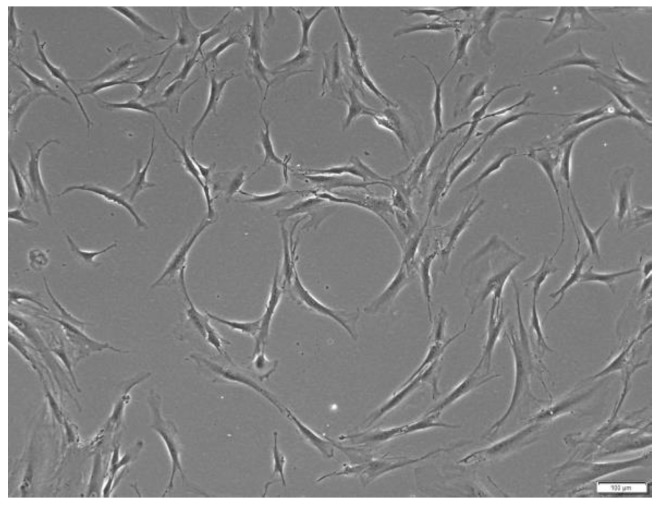
Representative image of HGF-1 cells cultured in DMEM medium supplemented with 10% fetal bovine serum before the exposure to HAp-based coatings. Scale bar: 100 µm.

The uncoated Si substrate and 5CeHAp-D and 10CeHAp-D composite coatings were sterilized by UV irradiation for 30 min and then placed in tissue culture plates. The human gingival fibroblasts (HGF-1 cell line purchased from American Type Culture Collection (ATCC), Cat. No. CRL-2014, Rockville, MD, USA) were seeded in 24-well culture plates (with or without 5CeHAp-D and 10CeHAp-D composite coatings) at a density of 5 × 10^4^ cells per well in Dulbecco’s Modified Eagle Medium (DMEM; Gibco/Invitrogen, Carlsbad, CA, USA), supplemented with 10% fetal bovine serum (FBS; Gibco/Invitrogen, Carlsbad, CA, USA), at 37 °C in a humidified atmosphere with 5% CO_2_ (Figure 2). After 24 h of cell exposure to HAp-based samples, several biocompatibility tests were performed. 

#### 2.5.2. Biocompatibility Assessment of CeHAp-D Composite Coatings

To test viable cell proliferation 3-(4,5-dimethylthiazol-2-yl)-2,5-diphenyltetrazolium bromide (MTT) assay was used. Authors chose lactate dehydrogenase (LDH) and MTT as good basic indicators to test cell viability and integrity, and chose nitric oxide (NO) as “a marker for inflammation”.

The cell viability was measured using the MTT assay, which is based on the quantification of NAD(P)H-dependent cellular oxidoreductase enzyme activity in the viable cells. After 24 h, the medium was aspirated, and the cultured fibroblasts were incubated with a 1 mg/mL MTT solution at 37 °C for 2 h. The purple formazan crystals formed in the metabolic active cells were dissolved with 2-propanol and the absorbance was measured at 595 nm using a GENiosTecan microplate reader (GENiosTecan, Salzburg, Austria).

The LDH amount released into the culture medium was determined as a measure of cell membrane integrity and cell viability using a commercial kit (Cytotoxicity Detection Kit-LDH, Roche, Basel, Switzerland) by reading the absorbance at 490 nm using a microplate reader (GENiosTecan, Salzburg, Austria). 

The level of NO released in the culture medium as an indicator of inflammation was assessed using the Griess reagent (a stoichiometric solution (*v*/*v*) of 0.1% naphthylethylendiamine dihydrochloride and 1% sulphanilamide in 5% H_3_PO_4_), after reading the absorbance at 550 nm.

In addition, the cell cytoskeleton morphology was visualized via fluorescence imaging, using cells fixed with 4% paraformaldehyde for 20 min and permeabilized with 0.1% Triton X-100—2% bovine serum albumin for 1 h. Filamentous actin (F-actin) was labeled with 20 μg/mL of phalloidin conjugated with fluorescein isothiocyanate (FITC) (Sigma-Aldrich, Munich, Germany) and the nuclei were counterstained with 2 μg/mL 4′,6-diamidino-2-phenylindole (DAPI) (Molecular Probes, Life Technologies, Carlsbad, CA, USA). Images were captured using an Olympus IX71 fluorescence microscope (Olympus, Tokyo, Japan).

#### 2.5.3. Statistical Analysis

The GraphPad Prism software (Version 9, GraphPad, San Diego, CA, USA), based on one-way ANOVA with Tukey’s multiple comparisons test, was used in order to conduct the statistical analysis of the obtained results in the revised manuscript. All data were expressed as mean value ± standard deviation (SD) of three independent experiments and a value of *p* < 0.05 was considered statistically significant.

## 3. Results and Discussion

**Figure 3:**
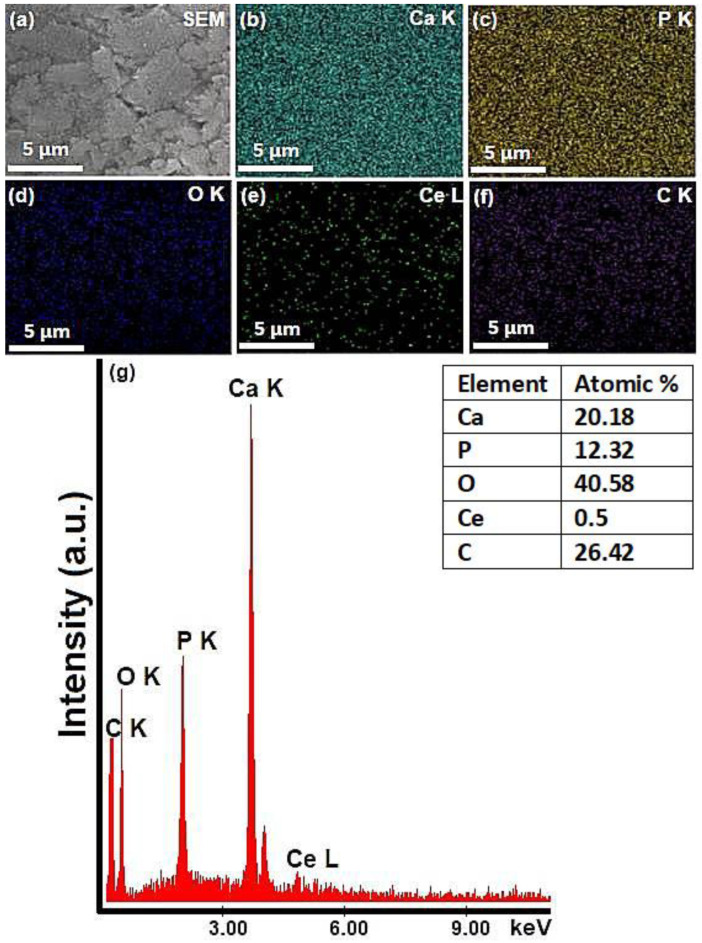
SEM image (**a**), EDS elemental mapping of the constituent elements of 5CeHAp-D composite target (**b**–**f**) and EDS spectrum of 5CeHAp-D composite target (**g**).

**Figure 4:**
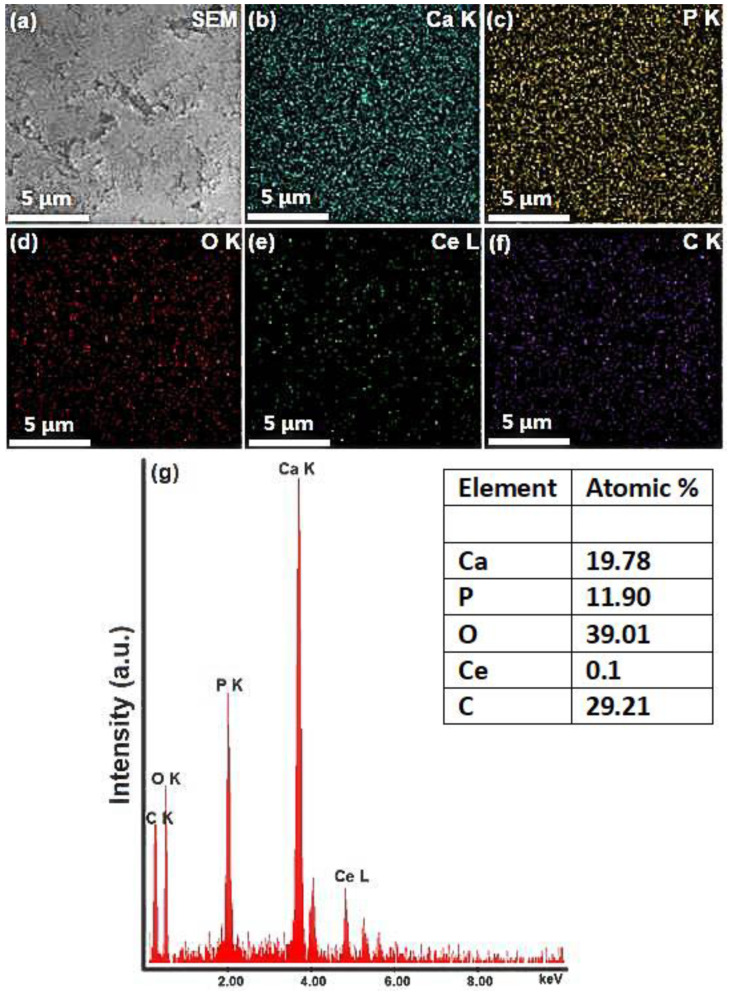
SEM image (**a**), EDS elemental mapping of the constituent elements of 10CeHAp-D composite target (**b**–**f**) and EDS spectrum of 10CeHAp-D composite target (**g**).

**Figure 5:**
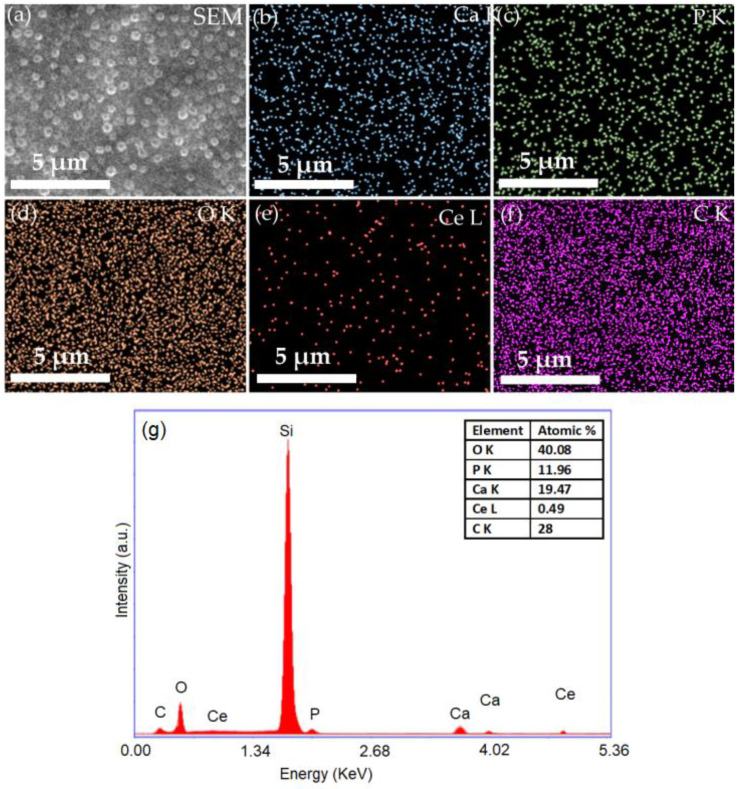
SEM image (**a**), EDS elemental mapping of the constituent elements of 5CeHAp-D composite coating (**b**–**f**) and EDS spectrum of 5CeHAp-D composite coatings (**g**).

**Figure 6:**
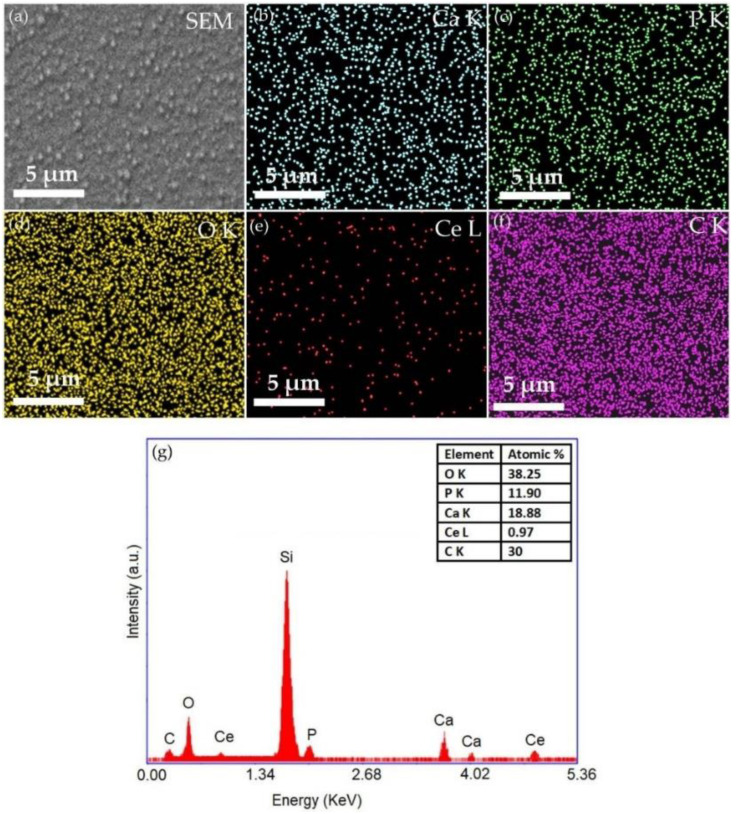
SEM image (**a**), EDS elemental mapping of the constituent elements of 10HApCe-D composite layer coating (**b**–**f**) and EDS spectrum of 10HApCe-D composite layer (**g**).

The morphological characteristics of 5CeHAp-D and 10CeHAp-D targets are presented in Figure 3 and Figure 4. Furthermore, information regarding chemical composition of the targets were obtained through EDS studies. The results of the EDS studies are also revealed in Figure 3 and Figure 4. Therefore, it can be noticed that the main chemical elements present in the chemical composition of the targets are Ca, P, O, Ce and C. All these elements belong to the 5CeHAp-D and 10CeHAp-D chemical compositions. Both in the EDS spectra and in the obtained cartographies presented in Figure 3 and Figure 4, the presence of impurities is not observed. Additionally, our results indicate the uniform and homogenous distribution of the main chemical constituents in the targets. The morphological features of the 5CeHAp-D and 10CeHAp-D composite coatings deposited on optically polished Si substrates were revealed after the scanning of their surfaces for various magnifications using SEM. Both films present granular structures on their surfaces (see Figure 5a and Figure 6a), while the layers are not cracked or exfoliated. In Figure 5a and Figure 6a the SEM images of the 5CeHAp-D and 10CeHAp-D composite thin films, with 5000× magnification, can be observed. Looking carefully at the SEM images from Figure 5a and Figure 6a, it can be observed that the granular structure formed on the surface of the 5HApCe-D composite layer is denser than in the case of the 10HApCe-D composite layer. We suppose that the embedding of cerium into the hydroxyapatite structure of the sputtering target and their simultaneous co-deposition by magnetron sputtering technique is responsible for these granular structures. More than that, the increase of the cerium concentration into the 10HApCe-D composite sputtering target, in comparison with the 5HApCe-D sputtering target, conduce to the generation into the plasma of a double number of Ce ions, which bombard the substrate during the deposition process. This conduces to a more compact granular structure of the 10HApCe-D composite layer than the 5HApCe-D composite layer. The size of the grain decreases from ~600nm (in 5HApCe-D composite layer) to ~300 nm (in 10HApCe-D composite layer).

Our targets preserve the HAp structure. EDS studies of thin films of CeHap-D have shown that the (Ca+Ce)/P ratio is slightly changed to 1.66, instead of 1.67 (for the targets). As a result of EDS evaluation of the thin films, we could say that the coatings have the structure of hydroxyapatite doped with cerium, coated with dextran, and slightly deficient in Ca.

The formation of these grain-like structures could reflect the columnar grain growth of the films. Crystalline HAp films with grain morphologies were obtained by Lopez et al. [29] in RF magnetron sputtering plasma in a substrate holder floating configuration. 

In our previous paper [7], when we deposited 5HAp-Ce layers on substrates placed on a grounded substrate holder in RF magnetron sputtering discharge, the grains were fine and hardly visible. Moreover, several studies [30,31] reported on the growth of columnar grains of calcium phosphate-based coatings, perpendicular to the grounded substrate, in RF magnetron sputtering discharges. The grain size depends on the plasma working conditions, such as gas pressure, deposition time, RF power, substrate holder–magnetron source distance and substrate bias. When the substrate is negative biased, the grain sizes of the coating are reduced due to the bombardment with ions of higher energy, thus favoring renucleation [30,31]. 

Another explanation of grain formation could be given by the simultaneous co-deposition of dextran and Ce doped hydroxyapatite. In our recent study [23], we have shown that coagulation of hydroxyapatite–chitosan macromolecules on the substrate surface conduce to nm grain-like structure formation. Formation of polymer nanoparticles is governed by the negative charges [23,32] accumulated on the substrate surface during the depositions. Firstly, the polymer vapors nucleate in nm size particles (on the substrate surface) that further coagulate into larger particles. 

The SEM–EDS elemental mapping of the 5CeHAp-D and 10CeHAp-D composite coatings, that corresponds to the images from Figure 5a and Figure 6a, are presented in Figure 5b–f and Figure 6b–f. The Ca, P, O, Ce and C elements are homogenously distributed over the entire analyzed areas. The EDS spectra (see Figure 5g and Figure 6g) of 5CeHAp-D and 10CeHAp-D composite coatings indicated that the Ce atomic percentage in the 10HApCe layer is higher than in the 5HApCe-D composite layer. The Ca K, P K, O K, Ce L and C K atomic percentages in both kinds of coating are shown in the tables from Figure 5g and Figure 6g.

The Ca/P ratio of calcium phosphate-based coatings depend on the RF magnetron plasma discharge working conditions [31,33] and substrate holder bias voltage. In RF magnetron discharge, the bombardment of the grounded substrates with negatively charged oxygen ions cause resputtering and removing of P, and PO_4_^3−^ groups from the deposited layers. Therefore, a decrease in the number of negative charged ions that reach the substrates could limit the sputtering of phosphate ions from the deposited layers. We suppose that the simultaneous co-deposition of Ce and HAp on floating substrates could explain the measured (Ca+Ce)/P ratio of about ~1.6. In our previous studies on RF magnetron plasma depositions of carbon phosphate coatings on grounded substrates, we determined a Ca/P ratio of about ~1.4 [24]. 

**Figure 7:**
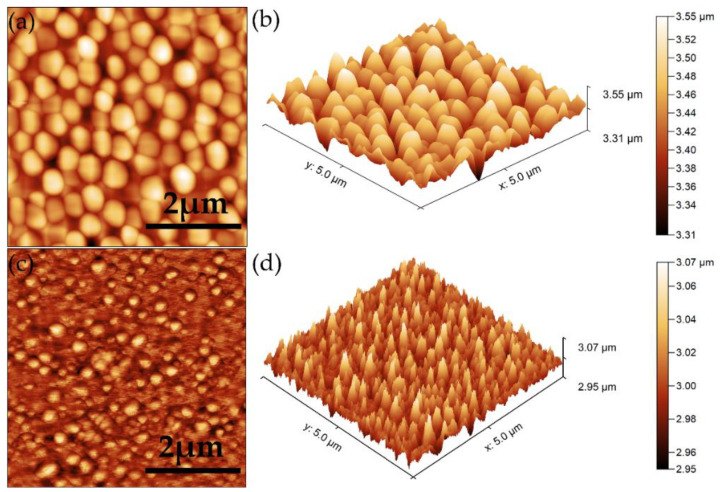
Typical 2D AFM images of surface topography obtained on 5CeHAp-D (**a**) and 10CeHAp-D (**c**) composite thin films and their 3D representation (**b**,**d**).

Atomic force microscopy was also used to obtain information about the surface morphology of the 5CeHAp-D and 10CeHAp-D composite thin films. The results depicting the AFM 2D surface topographies as well as their 3D representation are presented in Figure 7a–d. The 2D AFM micrographs and the 3D representation of the surfaces of the 5CeHAp-D and 10CeHAp-D composite thin films suggest the continuous and uniformly deposition of the layers. Moreover, the AFM topographies of the 5CeHAp-D and 10CeHAp-D composite thin films’ surfaces highlighted that the layers do not present any unevenness, fissures, or any other type of discontinuity. The root means square roughness (R_RMS_) parameters determined from the AFM topographies were R_Rms_ = 37.98 nm for 5CeHAp-D and R_Rms_ = 15.07 for 10CeHAp-D. The values obtained for the root mean square roughness imply that the surface topography of both samples is homogenous and does not present a significant roughness. On the other hand, the 2D AFM topographies, as well as their 3D representation, highlighted that the surface of the thin film is composed of nanostructured conglomerates, whose size decreased with the increase of the cerium ion concentration. These results are also in agreement with the SEM findings. 

**Figure 8:**
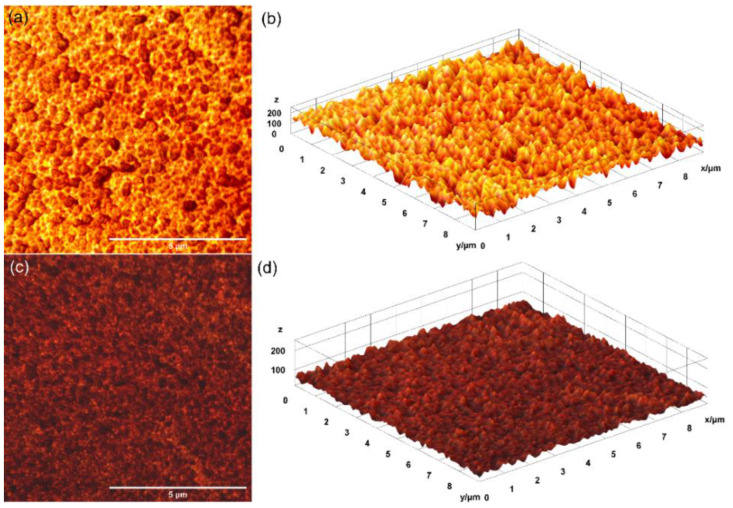
Metallographic microscopy image of the 5CeHAp-D (**a**) and 10CeHAp-D (**c**) composite thin films and their 3D representation (**b**,**d**).

Complementary information about the surface morphology of the thin films was obtained using metallographic microscopy. The 2D metallographic images of the 5CeHAp-D and 10CeHAp-D composite thin films, as well as their 3D representation, are depicted in Figure 8a–d. The 2D images were recorded using the 10× objective of the metallographic microscope and their 3D representation was achieved using ImageJ software [28]. The results of the metallographic microscopy investigations revealed that the surfaces of both the 5CeHAp-D and 10CeHAp-D composite thin films are homogenous and do not present any fissures, cracks, or discontinuities, having the general aspect of a continuous and uniformly deposited layer. Furthermore, the 2D and 3D images also suggested that the surfaces of the studied thin films are nanostructured. More than that, the results highlighted that the grain size of the structures decreased with the increase of the cerium concentration. 

**Figure 9:**
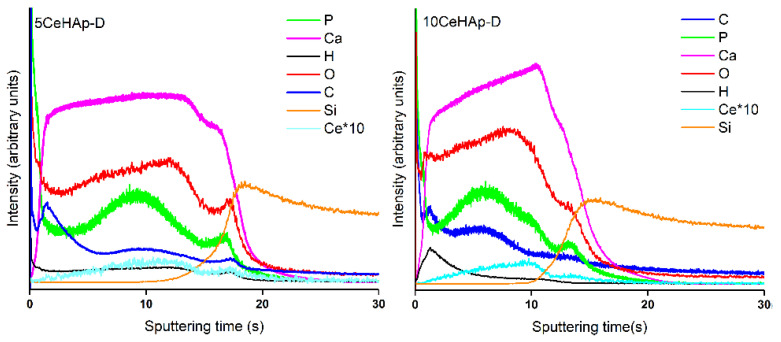
Compositional depth profiles of the 5CeHAp-D (**a**) and 10CeHAp-D (**b**) composite coatings obtained by GDOES.

Figure 9a,b present the depth profiles of the 5CeHAp-D and 10CeHAp-D composite thin films obtained by GDOES. By this technique, a large circular area of the layer (4 mm in diameter) is sputtered from its surface to the substrate in an RF discharge. The atoms contained in the sample are excited by inelastic collisions in the RF plasma and the emission line signals are recorded and processed to provide the elemental composition of the analyzed sample. Thus, the distribution of elements contained in the 5CeHAp-D and 10CeHAp-D layers, namely: Ca, P, O, C, H and Ce, were revealed, starting from the layer surface (t = 0 s) to the substrate interface as a function of the sputtering time. Due to the changes of sputtering rate during the GDOES depth profiling analysis, mainly at layer/substrate interfaces, the conversion of sputtering time into sputtered depth is not accurate and not recommended [34]. In Figure 9a,b, the layer/substrate interfaces are marked by the simultaneous decreasing of the intensities of the depth profile curves of the elements contained in the layer (Ca, P, C, O, H, Ce) and increasing of the intensities of the depth profile curves of the element characteristic to the substrate (Si).

The temporal evolution of Ca, P and Ce depth profile curves are similar (see Figure 9a,b), even at layer/substrate interface where some humps/peaks can be observed. These suggest the diffusion/implantation of Ca, P and Ce elements into the Si substrates as well as their linkages. Additionally, the sputtering time for the 10CeHAp-D sample is shorter than in the case of the 5CeHap-D sample, indicating a thinner 10CeHAp-D layer. These data are in agreement with SEM, AFM and metallographic microscopy analysis that shows a more compact structure of the 10CeHAp-D layer than of the 5CeHap-D layer.

The depth profile curves of C, O and H, which represent the chemical elements characteristic to dextran, are also observed in the graphs from Figure 9a,b. Their intensities are higher at shorter sputtering times (0–5 s) indicating the presence of a polymer, mainly at sample surfaces. These findings could sustain our assumption that the grains evidenced by the SEM images of the analyzed samples (see Figure 5a and Figure 6a) could also be attributed to the nano-sized structuring of dextran on layer surfaces.

**Figure 10:**
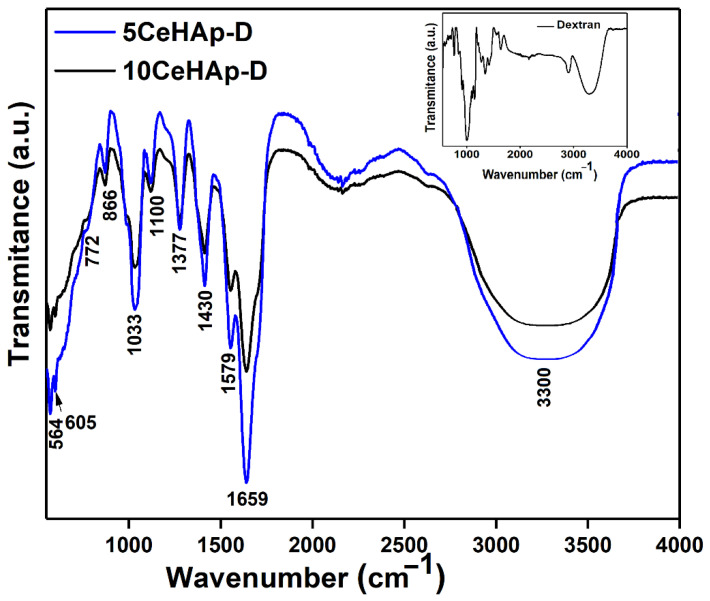
FTIR spectra of 5CeHAp−D and 10CeHAp−D composite thin films. The FTIR spectra of dextran is presented in the inset.

In Figure 10 the FTIR spectra obtained for both studied thin films are revealed. Mainly, the presence of the peaks related to the presence of ν_1_, ν_3_, and ν_4_ vibration modes of (PO_4_^3−^) groups from the hydroxyapatite (HAp) structure and to the specific vibration bands of the dextran structure could be noticed. Therefore, the main peaks that are noticed (in the FTIR spectra of 5CeHAp-D composite thin films) at around 564 cm^−1^, 604 cm^−1^, 961 cm^−1^, 1033 cm^−1^ and 1090 cm^−1^ are related to characteristic tetrahedral PO_4_^3−^ groups from HAp [26,35,36]. In the FTIR spectra of the 10CeHAp-D composite thin films, the main maxima are found at about 566 cm^−1^, 607 cm^−1^, 963 cm^−1^, 1035 cm^−1^ and 1092 cm^−1^ [26,35,36]. In the following section, we will only discuss the maxima obtained for the 5CeHAp-D composite thin films, with the maxima obtained for the 10CeHAp-D composite thin films being similar to these. The peak at around 564 cm^−1^ and 605 cm^−1^ corresponds to ν_4_ vibration mode of (PO_4_^3−^) groups [26,35,36]. Moreover, the vibrational bands observed at about 1033 cm^−1^ and 1090 cm^−1^ belongs to ν_3_ vibration mode of (PO_4_^3−^) groups from hydroxyapatite. In addition, the presence of the ν_1_ vibration mode of (PO_4_^3−^) groups at 961 cm^−1^ was observed, as in Figure 10 [26,35,36]. The main maxima that are characteristic to dextran structure could be noticed in the following spectral domains: 950–750 cm^−1^, 1200–950 cm^−1^ (due to C-O stretching), 1500–1200 cm^−1^ (due to symmetrical deformation of CH_2_ and C-OH deformations, respectively) [37,38]. Therefore, the maxima found at about 772, 1100, 1430 and 1377 cm^−1^ in the FTIR spectra are characteristic of polysaccharide (dextran) structures [37,38]. The main peak that is observed in the spectral region 3100–3500 cm^–1^ belongs to adsorbed water. All these peaks belong to the HAp and dextran structures without noting the presence of additional maxima specific to the presence of impurities in the analyzed samples. In the FTIR spectra of the 5CeHAp-D and 10CeHAp-D composite thin films (Figure 10), it could be noticed that by increasing the cerium concentration in the samples the obtained spectra are slightly displaced, at the same time a slight decrease in the intensity of the peaks was observed (with the increase of the cerium concentration in the samples). The results obtained in this research are thus in agreement with those previously reported in the literature [26,35,36].

**Figure 11:**
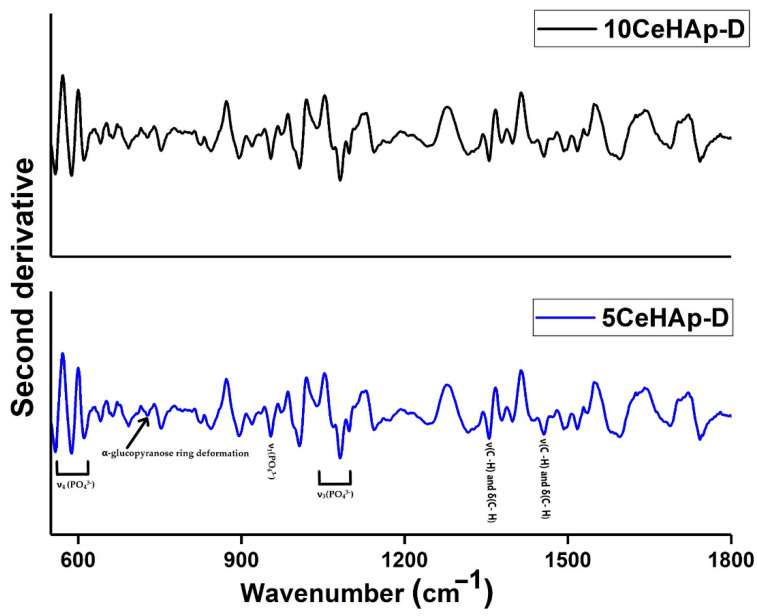
Second derivative spectra of 5CeHAp−D and 10CeHAp−D composite thin films.

For a comprehensive understanding of the experimental results obtained by FTIR studies, a second derivative analysis was performed. The results of the second derivative study performed in the 550–1800 cm^−1^ spectral domain are revealed in Figure 11. Our results clearly highlight the presence of maxima associated to ν_1_, ν_3_, and ν_4_ molecular vibration of (PO_4_^3−^) groups characteristic to HAp structure [26,39,40]. In addition, in the studied spectral domains the maxima associated with the characteristic vibration of dextran structures are revealed. In the spectral regions between 560–620 cm^−1^ and 900–1100 cm^−1^, respectively, are found the maxima that appear due to the presence of ν_1_, ν_3_, and ν_4_ vibration of (PO_4_^3−^) groups from the HAp structure [24,31,32]. Moreover, in Figure 11, the presence of the vibration bands specific to polysaccharides in the 950–750 cm^−1^, 1200–950 cm^−1^, 1500–1200 cm^−1^ spectral domains can be observed [37,38,41]. The maxima found around 1430 cm^−1^ and 1377 cm^−1^ belong to δ(C-H) and ν(C-H), meanwhile, the maxima found at 772 cm^−1^ belong to α-glucopyranose ring deformation [41]. 

**Figure 12:**
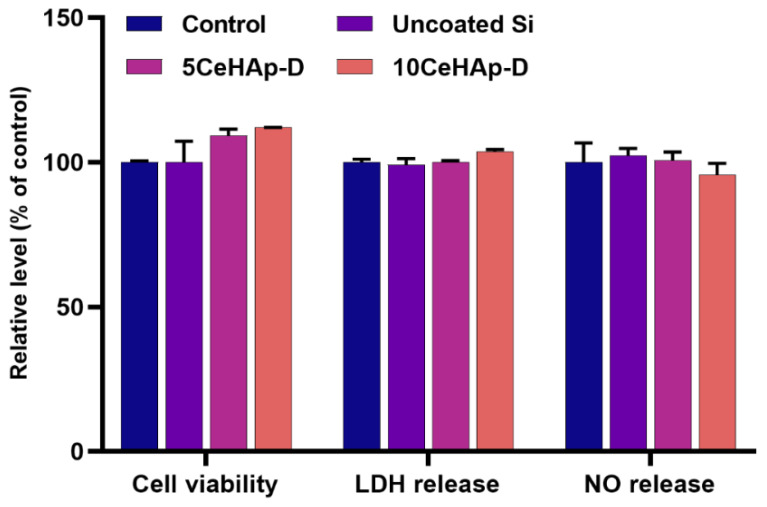
Biocompatibility of uncoated Si substrates and 5CeHAp-D and 10CeHAp-D composite coatings as shown by cell viability, lactate dehydrogenase (LDH), and nitric oxide (NO) release assays after 24 h exposure on normal gingival fibroblasts. Results are expressed as the mean ± standard deviation (SD) (*n* = 3) and represented relative to the untreated cells (control).

In vitro cell response to uncoated Si substrates and different coatings containing dextran coated cerium doped hydroxyapatite were tested on normal human gingival fibroblasts. For this purpose, cellular viability, membrane integrity and their potential to generate an inflammatory response were evaluated and the results are presented in Figure 12.

After 24 h of exposure to these bioactive coatings, the viability of gingival fibroblasts did not decrease but the small differences between the uncoated and the treated surfaces indicated that the best biocompatibility was exerted by the coatings containing dextran coated cerium doped hydroxyapatite. Moreover, the leakage of LDH into the cell culture medium was not recorded, demonstrating that none of the tested surfaces induced cell membrane permeabilization. These results were also confirmed by the NO release measurement that showed no significant changes between the controlled, uncoated Si surface and dextran coated cerium doped hydroxyapatite.

**Figure 13:**
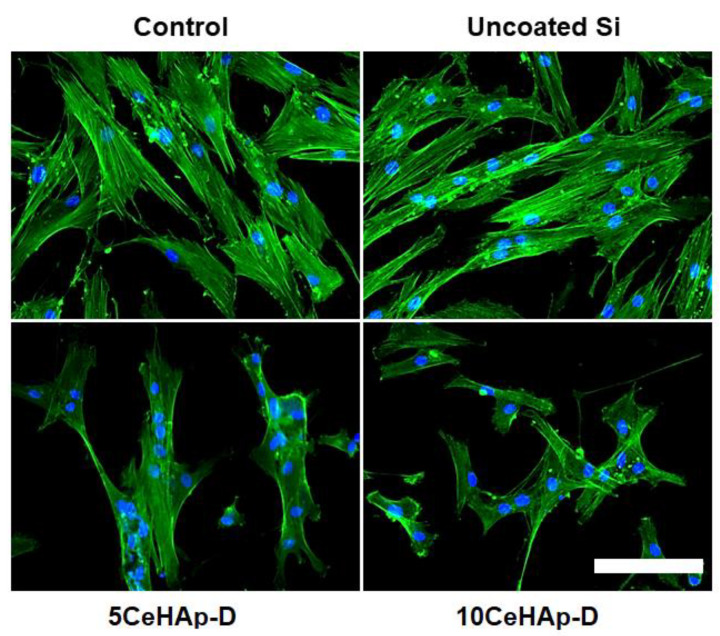
Actin cytoskeleton organization of gingival fibroblasts after 24 h of incubation with uncoated Si substrates and different dextran coated cerium doped hydroxyapatite coatings (5CeHAp-D and 10CeHAp-D). F-actin (green) was labeled with phalloidin-phalloidin-fluorescein isothiocyanate (FITC) and nuclei (blue) were counterstained with 4′,6-diamidino-2-phenylindole dihydrochloride (DAPI). Scale bar: 20 µm.

For a more in-depth characterization of the biological response induced by these modified Si surfaces in gingival fibroblasts, the morphology and actin cytoskeleton dynamics were also evidenced by fluorescence microscopy. The most representative images, as illustrated in Figure 13, were consistent with the results of the biocompatibility tests presented in Figure 12. Thus, it was shown that the human cells maintained their specific elongated morphology and established numerous focal adhesions after 24 h of incubation, which confirmed that the behavior of gingival fibroblasts and their proliferative capacity were not disturbed in the presence of the CeHAp-D composite coatings. These bioactive surfaces were harmless to the human gingival fibroblasts, proving good biocompatibility. 

**Figure 14:**
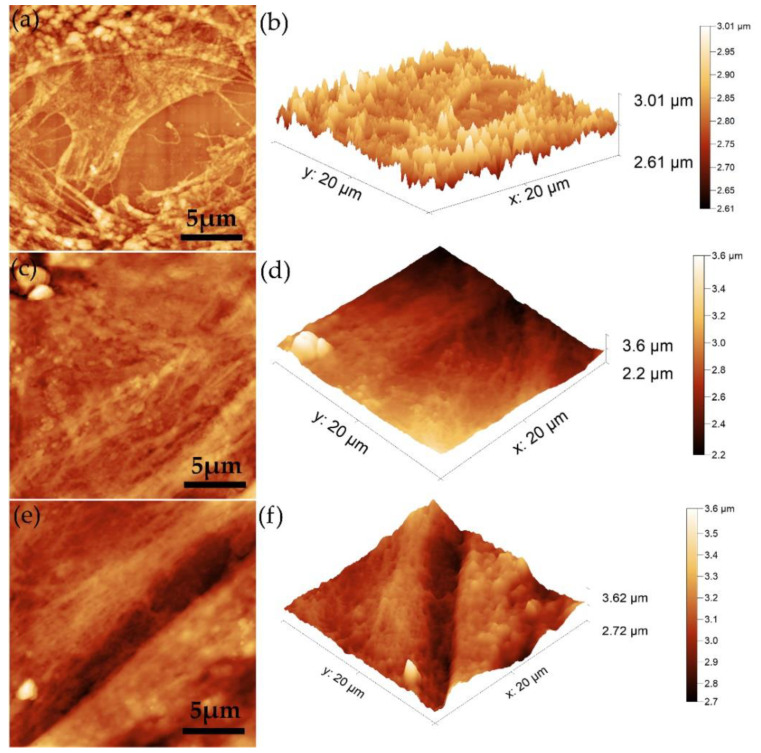
2D AFM topography of gingival fibroblasts after 24 h of incubation with uncoated Si substrates (**a**), 5CeHAp-D composite thin film (**c**) and 10CeHAp-D composite thin film (**e**) and their 3D representation (**b**,**d**,**f**).

Additional information regarding the adherence and biocompatibility of the 5CeHAp-D and 10CeHAp-D composite thin films was obtained using AFM. The AFM topographies of the thin films’ surfaces after being exposed with normal gingival fibroblasts were acquired at room temperature in normal atmospheric conditions on a surface of 20 × 20 µm^2^. The AFM topographies of the normal gingival fibroblasts’ adherence onto the surfaces of the uncoated Si disc and the 5CeHAp-D and 10CeHAp-D composite thin films are presented in Figure 14a–f. The 2D AFM topographies emphasized that on the surface of the investigated samples, typical patterns of the cellular morphology of normal gingival fibroblast cells could be seen, having typical flattened and elongated shapes [42,43,44,45]. Furthermore, both the 2D AFM topographies and their 3D representations highlighted that after an exposure of 24 h, the normal gingival fibroblasts exhibited a good adherence to the 5CeHAp-D and 10CeHAp-D composite thin films’ surfaces, and there is also clear evidence of their spread all over the thin films’ surfaces. On the other hand, the cells also adhered to the uncoated Si discs, but the AFM topographies revealed that they did not spread on the entirety of the Si discs’ surface. The AFM results demonstrated that normal gingival fibroblast cells spread equally and formed a monolayer that exhibited characteristic elongated fibroblastic morphology on the surface of the 5CeHAp-D and 10CeHAp-D composite layers. The AFM results are in agreement with the viability assays conducted and suggest that the CeHAp-D composite thin films (with x = 0.05 and 0.1) do not exhibit any cytotoxic effect against the normal gingival fibroblasts after 24 h of exposure, making them suitable for the future development of biomedical devices.

The present study sought to provide additional information on the behavior of dextran coated cerium doped hydroxyapatite thin layers, given that there is no other information in the existing literature. The composite material could have the properties of cerium (that stimulates the metabolic activity of organisms) [5], dextran (with a major role in reducing erythrocyte aggregation and platelet adhesion) and hydroxyapatite. The resulting composite material, thin layers of CeHAp-D, is a biocompatible and osteoconductive material with antibacterial activity [7]. The development of biocompatible CeHAp-D composite layers allowed us to obtain homogeneous coatings with compact granular structure and uniform distribution of constituent elements (Figure 5 and Figure 6). It was thus highlighted that the surfaces of the composite coatings do not show unevenness, cracks, or any other type of discontinuities (Figure 7 and Figure 8). Because the biological effects of cerium ions are not well known, gingival fibroblasts have been used in this research. This study proved that CeHAp-D biocomposite coatings can increase the viability of gingival fibroblasts. Furthermore, it has been established that the CeHAp-D biocomposite coatings did not induce cell membrane permeability. On the other hand, the measurement of NO release did not show significant changes between the control, the uncovered Si surface and the biocomposite coatings of CeHAp-D. This preliminary research also revealed that human cells maintained their specific elongated morphology and established numerous focal adhesions after 24 h of incubation (Figure 13 and Figure 14). It was demonstrated that CeHAp-D biocomposite coatings are harmless to human gingival fibroblasts, proving good biocompatibility. Thus, conducting complex biological studies to fully understand the influence of different parameters involved, such as homogeneity, thickness or roughness of layers, is justified in conducting future studies.

## 4. Conclusions

The radio frequency magnetron sputtering technique had the ability to produce CeHAp-D composite coatings (x = 0.05 and 0.1) on silicon substrate with homogenous surface structure and were harmless to the human gingival fibroblasts. The uniformity of CeHAp-D composite coatings was proved by SEM, AFM and MM investigations. The EDS and GDOES analysis confirmed the presence of constituent element of CeHAp-D into the coatings. The obtained CeHAp-D composite coatings were shown to be homogeneous. The presence of main vibrational peaks characteristic to HAp and dextran structure in the studied samples was highlighted by the results of FTIR studies. This study shows that the CeHAp-D composite coatings, obtained by radio frequency magnetron sputtering technique, have very good biocompatibility against human gingival fibroblasts. The fact that the 5CeHap-D and 10CeHAp-D composite coatings were harmless to human gingival fibroblasts shows that these films could be used successfully in medical applications. This study, through its results, proves to be essential in the effort to develop biocomposites with superior performance in medical applications.

## Data Availability

Not applicable.
